# Response of *Bacillus *cereus ATCC 14579 to challenges with sublethal concentrations of enterocin AS-48

**DOI:** 10.1186/1471-2180-9-227

**Published:** 2009-10-28

**Authors:** María J Grande Burgos, Ákos T Kovács, Aleksandra M Mirończuk, Hikmate Abriouel, Antonio Gálvez, Oscar P Kuipers

**Affiliations:** 1 Departamento de Ciencias de la Salud (Área de Microbiología), Facultad de Ciencias Experimentales, Universidad de Jaén, 23071-Jaén, Spain; 2 Department of Genetics, Groningen Biomolecular Sciences and Biotechnology Institute, University of Groningen, Kerklaan 30, 9751NN Haren, The Netherlands; 3 Kluyver Centre for Genomics of Industrial Fermentation, Delft/Groningen, The Netherlands

## Abstract

**Background:**

Enterocin AS-48 is produced by *Enterococcus faecalis *S48 to compete with other bacteria in their environment. Due to its activity against various Gram positive and some Gram negative bacteria it has clear potential for use as a food preservative. Here, we studied the effect of enterocin AS-48 challenges on vegetative cells of *Bacillus cereus *ATCC 14579 by use of transcriptome analysis.

**Results:**

Of the 5200 genes analysed, expression of 24 genes was found to change significantly after a 30 min treatment with a subinhibitory bacteriocin concentration of 0.5 μg/ml. Most of up-regulated genes encode membrane-associated or secreted proteins with putative transmembrane segments or signal sequences, respectively. One operon involved in arginine metabolism was significantly downregulated. The BC4206-BC4207 operon was found to be the most upregulated target in our experiments. BC4206 codes for a PadR type transcriptional regulator, while BC4207 codes for a hypothetical membrane protein. The operon structure and genes are conserved in *B. cereus *and *B. thuringiensis *species, but are not present in *B. anthracis *and *B. subtilis*. Using real-time qPCR, we show that these genes are upregulated when we treated the cells with AS-48, but not upon nisin treatment. Upon overexpression of BC4207 in *B. cereus*, we observed an increased resistance against AS-48. Expression of BC4207 in *B. subtilis *168, which lacks this operon also showed increased resistance against AS-48.

**Conclusion:**

BC4207 membrane protein is involved in the resistance mechanism of *B. cereus *cells against AS-48.

## Background

*Bacillus cereus *is a Gram positive rod-shaped aerobic, endospore-forming bacterium. Strains of *B. cereus *are widely distributed in the environment, mainly in soil, from where they easily spread to many types of foods, especially of vegetable origin, as well as meat, eggs, milk, and dairy products. This bacterium is one of the leading causes of food poisoning in the developed world. *B. cereus *causes two types of food-borne intoxications. One type is characterized by nausea and vomiting and abdominal cramps and has an incubation period of 1 to 6 hours. This is the "short-incubation" or emetic form of the disease. The second type is manifested primarily by abdominal cramps and diarrhea with an incubation period of 8 to 16 hours. This type is referred to as the "long-incubation" or diarrheal form of the disease [1,2].

Different strategies may be employed to prevent *B. cereus *poisoning, like heating food above 75°C before use to kill vegetative cells. However, increasing trends for use of packed foods require new food preservation methods to increase the safety levels against *B. cereus*. One of the current approaches is the use of antimicrobial peptides (either alone or in combination with other hurdles) such as enterocin AS-48 and other bacteriocins [3-5].

Bacteriocins are small, ribosomally-synthesized antimicrobial peptides synthesized and used by one bacterium as to inhibit growth of similar or closely related bacterial strains [6]. Bacteriocins are categorized in several ways, e.g. on basis of the producing strain, common resistance mechanisms, and mechanism of killing. Enterocin AS-48 is a broad-spectrum antimicrobial peptide produced by *Enterococcus faecalis *S-48, belonging to Class III of enterococcal bacteriocins or enterocins [7]. Enterocin AS-48 is a 70-residue cyclic peptide with a molecular weight of 7.15 kDa [8]. The crystal structure of enterocin AS-48 has been resolved to 1.4 Ǻ resolution [9]. It is unique with respect to its natural cyclic structure in which N and C termini are linked by a peptide bond. It has been shown that enterocin AS-48 adopts different oligomeric structures according to physiochemical conditions: it exists in monomeric form at pH below 3 and in dimeric form in the pH range of 4.5 to 8.5. The molecules of AS-48 in the crystal are arranged in chains of pairs of molecules linked either by hydrophobic interactions (dimeric form I, abbreviated to DF-I), or by hydrophilic interactions (dimeric form II, abbreviated to DF-II). The molecules within the DF-I interact through the hydrophobic helices H1 and H2. On the other hand, the hydrophilic surfaces of helices H4 and H5 are interacting in DF-II.

The mode of action of enterocin AS-48 has been elucidated [10]. This bacteriocin makes pores of an approximate size of 0.7 nm in the bacterial cytoplasmic membrane thereby disrupting the proton motive force and causing cell death [10]. It also shows a secondary, bacteriolytic effect against some of the target bacteria. Based on its crystal structure, the proposed mechanism of action suggests that the two different stages of molecular association, DF-I and DF-II, are involved in changing from the water-soluble DF-I to the membrane-bound DF-II stage at the membrane surface. This transition implies a 90° rotation of each protomer within DF-I, in a way that the partially hidden hydrophobic helices H1 and H2 become solvent accessible [9]. This would permit AS-48 to insert into the bacterial membrane.

Although the mechanism of action of enterocin AS-48 has been studied extensively at physiological and physico-chemical levels, nothing is known about the responses of sensitive bacterial cells upon exposure to the bacteriocin. Previous experiment in our laboratory with AS-48 against *Listeria monocytogenes *showed that bacterial cells can be adapted to AS-48, thereby increasing resistance against AS-48 [11]. This adaptation can be achieved with subsequent inoculation in the presence of low, but still inhibitory concentrations of AS-48. However, the adaptation is gradually lost upon repeated subcultivation. Given the great interest of enterocin AS-48 as a food preservative, it is of high relevance to know how the target bacteria react to bacteriocin treatment. This may have direct implications on the elucidation of probable mechanisms for cell adaptation as well as the development of bacteriocin resistance mechanisms. Moreover, a better knowledge of the bacterial response to enterocin AS-48 may also allow identification of new targets that could be exploited to enhance bacteriocin activity. The purpose of the present study was to determine the genome-wide response of *B. cereus *cells exposed to enterocin AS-48 and to identify components that help the bacterium to survive bacteriocin treatments.

## Results

### Effect of enterocin AS-48 on global gene expression in *B. cereus *ATCC14579

**Figure 1:**
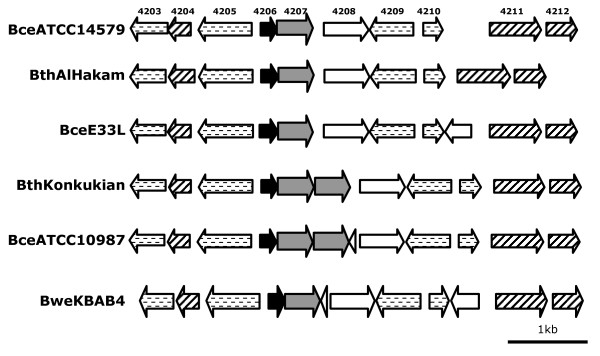
**Conservation of BC4206-BC4207 operon and surrounding genes in fully sequenced *B. cereus, B. thuringiensis *and *B. weihenstephanensis *species**. BC4203-BC4212 numbers are depicted as genes are labelled in the genome of *B. cereus *ATCC14579. Arrows indicate the BC4207 homologue (in grey), PadR homologues (in black), conserved proteins with putative function: BC4203, BC4205, BC4209 and BC4210 encoding for putative hydrolase, spore lyase, lypoate-protA ligase and rhodase, respectively (dashed lined), putative conserved regulators: BC4204, BC4211 and BC4212 for putative iron dependent repressor, LacI type regulator and TetR like regulator, respectively (stripped) and other putative genes (in white).

Enterocin AS-48 was shown to inhibit growth of vegetative cells and spore outgrowth of *B. cereus *[12] and it can be an effective bioagent against *B. cereus *in various food related media, e.g. hard cheese, rice based foods, fruit and vegetable juices [13-15]. Although the mode of action of AS-48 is well understood, the response of bacteria to enterocin AS-48 is poorly examined. We have therefore determined the transcriptome of *B. cereus *ATCC14579 in response to AS-48. To omit the effect of growth inhibition related differences between the treated and the control culture, a subinhibitory bacteriocin concentration of 0.5 μg/ml of AS-48 was used in our experiments. We observed no adaptation process, when *B. cereus *was subsequently cultivated in the presence of 0.5 μg/ml of AS-48, but only when cells were treated with low, but inhibitory concentration of AS-48 (data not shown). In preliminary experiments samples were taken 15 and 30 min after addition of AS-48, but we could not detect any significant changes after 15 min (data not shown), therefore final transcriptome comparisons were performed on samples incubated with AS-48 for 30 min. Transcriptome analyses were performed on six independent biological replicates. 20 genes were identified to be significantly upregulated, and only 4 genes to be downregulated (Table 1). All 4 downregulated genes (BC0406-BC0409) are located in one putative operon, coding for proteins involved in arginine and proline metabolism. Most of the upregulated genes code for proteins located in the membrane (highlighted in bold in Table 1) or contain a signal sequence or periplasmic domain. The putative membrane proteins show similarity to different transport or permease proteins or have been annotated as a hypothetical protein with no known function. Two regulators belonging to the PadR family were significantly upregulated, both located upstream of, and in one operon with, genes coding for membrane proteins that are similarly enhanced during our experiments. Here, we selected the most upregulated putative operon (BC4206-4207) for further characterization. The BC4206-4207 operon is conserved in all fully sequenced *B. cereus*, *B thuringiensis *and *B. weihenstephanensis *genomes except in the *B. cereus *Cytotoxis strain, but is missing in *B. anthracis *and other *Bacillus *species. When this operon is found in a genome, the genes surrounding this operon are also conserved (Figure 1).

**Table 1:** Summary of transcriptional changes in *B. cereus *ATCC14579 upon 0.5 μg/ml AS-48 treatment

Locus tag	Expression ratio^a^	Significance (p-value)^b^	Annotation^c^	Feature^d^
**Upregulated**				

BC4206	8.7	< 10^-14^	PadR-like transcriptional regulator	PR
**BC4207**	**8.7**	**< 10^-14^**	**Hypothetical protein**	**TMS(4)**
**BC4027**	**4.7**	**< 10^-14^**	**NADH dehydrogenase subunit N**	**TMS(6)**
**BC2842**	**4.0**	**10^-12^**	**Hypothetical protein**	**SS; TMS(2)**
**BC5438**	**3.7**	**10^-12^**	**Antiholin-like protein**	**TMS(7)**
**BC1612**	**3.7**	**10^-13^**	**Na+/H+ antiporter**	**SS; TMS (11)**
**BC2300**	**3.0**	**10^-11^**	**Oxalate/formate antiporter**	**SS; TMS(11)**
**BC5439**	**2.7**	**10^-10^**	**Murein hydrolase regulator**	**SS; TMS(4)**
BC4528	2.4	10^-11^	Ferrichrome-binding protein	PPD
**BC4028**	**2.4**	**10^-10^**	**NADH dehydrogenase subunit N**	**TMS(6)**
**BC4268**	**2.4**	**10^-8^**	**Phosphate transport system permease protein**	**TMS(6)**
BC4269	2.3	10^-9^	Phosphate-binding protein	SS
**BC4362**	**2.2**	**10^-7^**	**Ferrichrome transport system permease protein**	**TMS(9)**
**BC0223**	**2.2**	**10^-9^**	**Hypothetical protein**	**SS; TMS(1)**
BC4029	2.2	10^-11^	PadR-like transcriptional regulator	PR
**BC5100**	**2.1**	**10^-8^**	**Hypothetical protein**	**SS; TMS(1)**
BC0383	2.1	10^-10^	Ferrichrome-binding protein	SS, PPD
BC3540	2.1	10^-9^	BNR-repeat containing protein	
BC3541	2.1	10^-7^	Flavodoxin	Flavodoxin
**BC0227**	**2.0**	**10^-9^**	**Hypothetical protein**	**TMS(1)**

**Downregulated**				

BC0409	0.3	10^-12^	Carbamate kinase	Kinase
BC0406	0.3	10^-12^	Arginine deiminase	Aminidotransferase
BC0407	0.3	10^-12^	Ornithine carbamoyltransferase	Carbamoyl-P binding domain; Asp/Orn binding domain
**BC0408**	**0.3**	**10^-12^**	**Arginine/ornithine antiporter**	**Permease; TMS(1)**

^a ^The ratio of gene expression is shown. Ratio: expression in AS-48 treated sample over that in untreated samples.

^b ^Bayesian *p *value

^c ^Putative function of protein as annotated in the *B. cereus *ATCC14579 genome sequence

^d ^Domains detected using SMART search http://smart.embl-heidelberg.de/ (Letunic et al., 2006 Nucl Acid Res 34: D257-D260). PR; PadR domain; SS, signal sequence; TMS(n), transmembrane segment (n is the number of such domain); PPD, periplasm domain.

Proteins with TMS are highlighted in bold

### Validation of array experiments

**Figure 2:**
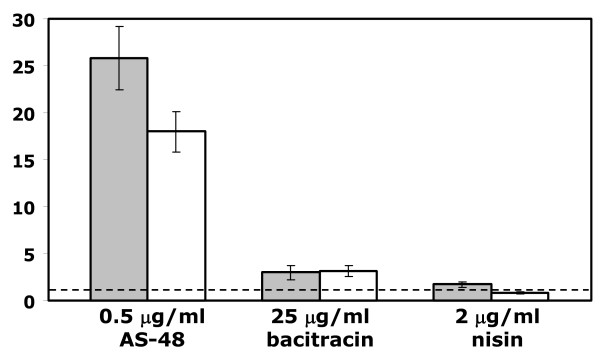
**RT-qPCR detection of *B. cereus *BC4207 and BC4028 genes**. Relative expressions of BC4207 (grey bars) and BC4028 (white bars) were determined in AS-48, bacitracin and nisin treated *B. cereus *ATCC14579 cultures (see Methods for concentrations). Transcript levels of genes were normalized to the level of house keeping *rpoA *gene and compared to untreated samples (dashed line).

Real-time RT-PCR was performed on independent samples to validate our array results. To verify that upregulation of the genes were the result of specific treatment with AS-48 and not a general response, we also applied samples that were incubated in the presence of sublethal amount of bacitracin (25 μg/ml) or nisin (2 μg/ml), bacteriocins that both affect cell wall biosynthesis through blocking the lipid II cycle by interaction with C55-isoprenyl pyrophosphate [16] or forming pores in cell membrane during interaction with lipid II [17,18], respectively. Two genes were selected (BC4207 and BC4028), both coding for a putative membrane protein and both located downstream of a PadR like regulator (BC4206 and BC4029, respectively), for quantitative real time RT-PCR. Quantitative real time PCR showed 26 ± 6 and 18 ± 4 times upregulation of BC4207 and BC4028 in samples treated with AS-48 compared with control samples, respectively (Figure 2). Similar analysis of samples treated with AS-48 for 15 min showed less then 2 times induction of the BC4207 and BC4028 genes, in agreement with the lack of significant changes after 15 min of AS-48 treatment in microarray experiments. Samples incubated in the presence of bacitracin showed slightly enhanced expression of target genes, while addition of nisin did not significantly change the transcription of these genes.

### Overexpression of BC4207 increases resistance against AS-48 in *B. cereus *and *B. subtilis*

To resolve whether increased expression of the putative membrane protein BC4207 helps *B. cereus *to defend itself against AS-48, BC4207 was cloned behind the IPTG inducible Pspac promoter and expression was induced in *B. cereus *ATCC14579. After preliminary induction of *B. cereus *containing pATK33 using 1 mM of IPTG, cells were exposed to varying amounts of AS-48 and growth was followed in time. As depicted in Table 2, cells containing overexpressed BC4207 were able to survive in the presence of slightly increased amounts of AS-48, compared to cultures containing control plasmid pLM5 or when BC4207 was not induced. Important to note is that BC4207 is already expressed in wild type *B. cereus *in response to AS-48 explaining the relatively low level of increased resistance upon further overexpression of BC4207. Unfortunately, we were not able to obtain a knockout of BC4207 to show the expected increased sensitivity. To support the idea that the increased resistance of *B. cereus *cells against AS-48 is caused by specific overexpression of the BC4207 membrane protein, we randomly selected two membrane proteins (BC4147 and BC4744) and introduced them into *B. cereus *ATCC14579 similar to the BC4207 protein. Expression of these proteins resulted in no significant growth difference in the presence of various amounts of AS-48 compared to the strain containing the pLM5 control plasmid. Further, comparative transcriptome analyses of *B. cereus *carrying pLM5 control plasmid and the BC4207 overexpressing plasmid pATK33 in the presence of IPTG revealed the significant (p-value < 10^-5^) upregulation of the BC4207 gene (13.6 fold) and downregulation of the BC5171 and BC5073 genes (11.6 fold and 9.3 fold, respectively), when BC4207 was expressed (data not shown). *B. cereus *containing pATK33 was challenged with bacitracin and nisin, but expression of BC4207 did not change the resistance of *B. cereus *against these bacteriocins (data not shown).

**Table 2:** Growth inhibition of *B. cereus *ATCC14579 and *B. subtilis *168 strains containing BC4207 expression plasmid pATK33 or control plasmid pLM5 in the presence of various AS-48 concentrations.

Strain		IPTG^a^	MIC^b^
*B. cereus *ATCC14579	pLM5	-	2.5
		**+**	**2.5**
	pATK33	-	2.5
		**+**	4.5*
*B. subtilis *168	pLM5	-	1.0
		**+**	**1.0**
	pATK33	-	1.5
		**+**	5.0*

(^a^) Cells were growth in the absence (-) or presence (+) of IPTG (bold).

(^b^) Minimal inhibitory concentrations are given in μg/ml of AS-48.

* p-value < 0.005; > 6 cultures as determined with Student's t-test.

No gene coding for a BC4207 homologue can be identified in the fully sequenced genome of *B. subtilis *168. BC4207 was introduced and expressed in *B. subtilis *with a similar method used for *B. cereus*. Upon induction of BC4207 the sensitivity of *B. subtilis *was diminished against AS-48. LiaRS was previously reported to respond to cell envelope stress and the target gene *liaI *was highly upregulated by LiaR in response to the addition of bacitracin or nisin to the medium [19]. To see, whether the presence of AS-48 had similar effects on the expression of the *liaI *gene, we have assayed the expression form *liaI *promoter in response to the addition of AS-48. In contrast to the high upregulation of *liaI *in the presence of bacitracin, we have observed no induction of expression when bacteria were incubated with various amount of AS-48 (data not shown).

## Discussion

In the present study a sublethal AS-48 concentration was used to detect gene expression differences in *B. cereus *ATCC14579 that result from interaction of AS-48 with the cells, but not in response to cell death induced by AS-48. We aimed to determine which genes help *B. cereus *to survive confrontation with AS-48 and identify possible resistance mechanisms. While there was very mild change in the growth after 30 min. incubation with a sublethal bacteriocin concentration, at least 24 genes were affected significantly (Table 1). The observed changes in gene expression were mostly related to up-regulation of membrane associated or periplasmic proteins and downregulation of an operon involved in arginine/ornithine catabolism. Downregulation of argnine/ornithine metabolic genes might be related to the slight difference in growth upon AS-48 treatment that is not apparent using OD measurements. Also, this downregulation might cause a change in local pH at the cell wall in view of the decreased catabolic production of NH_3 _and CO_2_. Upregulated genes coded for hypothetical membrane proteins or putative transporters. The BC4206-BC4207 operon was most heavily upregulated in *B. cereus *upon AS-48 treatment. BC4206 is PadR type regulator, while BC4207 is a hypothetical membrane protein with 4 transmembrane segments. Members of the PadR family are known to have a function in regulating cellular pathways resulting in multidrug resistance, virulence or detoxification [20,21]. These proteins involved in resistance mechanisms, are generally encoded in the vicinity of the *padR *genes. Overexpression of the BC4207 protein in both *B. cereus *and *B. subtilis *results in elevated resistance against AS-48. Upon overexpression of BC4207, we have found no other genes to be upregulated (data not shown), suggesting that increase in BC4207 expression alone raised the resistance of *B. cereus *against AS-48. Interestingly, enhanced resistance upon BC4207 overexpression was specific to enterocin AS-48 and not observed in the presence of bacitracin or nisin. Bacitracin and nisin both effect cell wall biosynthesis through blocking the lipid II cycle [16] and forming pores in the cell membrane during interaction with lipid II [17,18], respectively. This is not the case of enterocin AS-48, since the primary action of this antimicrobial peptide, like most other bacteriocins, is the disruption of the cytoplasmic membrane.

In spite of recent advances on genome and transcriptome analysis, there are very few reports on the effects of antimicrobial substances on bacterial gene expression. Recently, Martínez et al. (2007) [22] reported that lactococcin 972 (Lcn972) significantly upregulated the expression of 26 genes in *Lactococcus lactis*, most of which encode membrane proteins of unknown function and the two component system (TCS) CesSR (formerly known as TCS-D) associated with cell-envelope stress. Lcn972 is a non pore-forming bacteriocin that inhibits the synthesis of peptidoglycan at the septum in *Lactococcus lactis*. Moreover, the response of a number of Gram-positive bacterial species towards cell wall active antibiotics has been studied recently by using genome-wide transcription analysis [19,23-27]. Essentially, these reports describe a very complex system involving the concerted action of extracellular sigma factors and two-component systems (TCSs) [28]. LiaRS, the *B. subtilis *homologue of CesSR, was unable to activate *liaI *expression in *B. subtilis *in response to AS-48 treatment. Therefore, the effect of AS-48 on bacterial gene expression clearly differs from the mechanisms described earlier for *B. subtilis *[28].

The precise way in which BC4206 responds to the presence of AS-48 needs to be deciphered by further experimental work, including determining the target genes of BC4206 and the exact signal sensed by this PadR-type regulator. The structure and function of the BC4207 membrane protein and its role in the resistance mechanism against AS-48 is also particularly intriguing and target of our future research.

## Conclusion

*B. cereus *cells, when treated with bacteriocin AS-48, increase the expression of the BC4207 gene coding for a putative membrane protein. Targeted inactivation of the BC4207 protein might be useful to increase the effect of AS-48 on food poisoning *B. cereus *cells.

## Methods

### Bacterial strains, growth conditions and preparation of cells for RNA isolation

*Bacillus cereus *ATCC 14579 and *B. subtilis *168 strains from glycerol stocks were grown overnight on TY broth at 30°C, with shaking at 225 rpm. Cultures were diluted to a final OD_600 _of 0.15 in fresh TY medium. *B. cereus *ATCC14579 and *B. subtilis *168 strains containing pATK33 or pLM5 were grown in the presence of 50 and 10 μg/ml of kanamycin, respectively.

Growth of *B. cereus *and *B. subtilis *in the presence of various concentration of bacteriocin was monitored every 15 minutes using a TECAN GENios Absorbance Reader (TECAN).

When cultures reached an OD_600 _of 0.3, purified enterocin AS-48 was added to the cultures at a concentration of 0.5 μg/ml, which was the maximal concentration not inhibiting growth, cells were harvested after 15 or 30 min by centrifugation and cell pellets were immediately frozen in liquid nitrogen and stored at -80°C until RNA isolation. Six independent biological replicates were used for microarray analysis. For quantitative RT-PCR, cells were treated with nisin and bacitracin at a subinhibitory concentration of 2 μg/ml and 25 μg/ml, respectively.

### Purification of AS-48

Enterocin AS-48 was purified to homogeneity by reversed-phase high-performance chromatography as described elsewhere [29].

### RNA isolation

RNA extraction was performed with the Macaloid/Roche method [30] with 2 additional steps of phenol-chloroform washing. Purified RNA concentration was measured using a Nanodrop spectrophotometer at 260 nm. The quality of purified RNA was checked with a 50 ng/μl sample by using a BioAnalyser.

### DNA-microarray analysis

DNA-microarrays containing amplicons of 5200 annotated genes in the genome of *B. cereus *ATCC 14579 were designed and produced as described previously [31]. Slide spotting, slide treatment after spotting, and slide quality control were performed as described elsewhere [30].

Data were analysed essentially as described before [32]. Each ORF is represented by duplicate spots on the array. After hybridization, fluorescent signals were quantified with the ArrayPro analyser, and processed with Micro-Prep [31]. Statistical analysis was performed using CyberT [33]. Genes with a Bayes P-value below 1.0 × 10^-4 ^with at least twofold differential expression were considered to be significantly affected. Microarray data has been deposited in Gene Expression Omnibus database (GSM412591).

### Quantitative RT-PCR

Following RNA purification, samples were treated with RNase-free DNase I (Fermentas) for 60 min at 37°C in DNaseI buffer (10 mmol·l^-1 ^Tris·HCl (pH7.5), 2.5 mmol·l^-1 ^MgCl_2_, 0.1 mmol·l^-1 ^CaCl_2_). Samples were purified with the Roche RNA isolation Kit. Reverse transcription was performed with 50 pmol random nonamers on 1 μg of total RNA using RevertAid™ H Minus M-MuLV Reverse Transcriptase (Fermentas). Quantification of cDNA was performed on an iCycler iQ (BioRad) using iQ SYBR Green Supermix. The following primers were used: for BC4207, qBCE5 (5'-GAGCAACAAATGGAAGAACTG-3') and qBCE6 (5'-TGTTTGAGTTGGTAAAGCTG-3'), for BC4028 qBCE7 (5'-CTCCATTTAATTGAGGGTGAG-3') and qBCE8 (5'-GTTTCCTGTCTATCTCTTTCCA-3') and for rpoA gene of *B. cereus*, qBCE3 (5'-CGTGGATATGGTACTACTTTGG-3') and qBCE4 (5'-TTCTACTACGCCCTCAACTG-3'). The amount of BC4207 and BC4028 cDNA was normalized to the level of *rpoA *cDNA using the 2^-ΔΔCt ^method [34].

### Overexpression of the BC4207, BC4147 and BC4744 proteins

BC4207, BC4147 and BC4744 genes were amplified with oMJGB3 (5'-GATCGAAGCTTACGGTAAATAACTTATTACAG-3') and oMJGB4 (5'-GATCCAGGCATGCTCACGTCAACAATTAACTTT-3'), oBCE9 (5'-CATATAGGAGTAATGATATG-3') and oBCE10 (5'-AGAGAAGATACGGCATAG-3'), oBCE11 (5'-TACAAGGAGTTGCTTTATGG-3') and oBCE11 (5'-TTATATCGGCGCAACTAC-3'), respectively. PCR products were cloned into the Eco47III site of pLM5 vector [35], resulting in pATK33, pATK49 and pATK411, respectively. Plasmids were introduced into the *B. cereus *ATCC14579 and *B. subtilis *168 strains by electroporation [36] and natural transformation [37], respectively. IPTG was used at a final concentration of 1 mM to induce the overexpression of proteins.

### Biological activity

Antimicrobial activities of bacteriocins were determined as minimal inhibitory concentration (MIC) values against various *Bacilli *following previous practice [38]. Growth was followed in the presence of various concentrations of bacteriocin and monitored every 15 minutes using a TECAN GENios Absorbance Reader (TECAN). Without bacteriocin addition, the final OD_600 _values were about 1.1 ± 0.1 for *B. cereus*, 0.8 ± 0.1 for *B. subtilis*. All experiments were performed in triplicate. Inhibition curves were made by plotting OD_600 _at the end of exponential phase in the case of the non treated strain versus bacteriocin concentration. The minimal inhibitory concentration values were determined from the inhibition curves by interpolation. The lowest concentration of bacteriocin at which less than 1% of the total increase in the OD_600_, measured in the absence of bacteriocin, had occurred, was taken as the MIC value. The MIC values of AS-48, nisin and bacitracin for *B. cereus *ATCC14579 were 2.5 μg/ml, 5 μg/ml and 50 μg/ml, respectively.

## Authors' contributions

MJGB and ATK contributed equally to this work. MJGB carried out the microarray experiments and wrote the manuscript, ATK performed the quantitative RT-PCRs and overexpression of BC4207 and was involved in writing the manuscript, AMM participated in the design of the growth assay and microarray experiments, AH helped to obtain the purified AS-48 bacteriocin, AG and OPK conceived and coordinated the project, and corrected the manuscript. All authors have read and approved the manuscript.
